# Circulating blood biomarkers correlated with the prognosis of advanced triple negative breast cancer

**DOI:** 10.1186/s12905-023-02871-6

**Published:** 2024-01-13

**Authors:** Xingyu Li, Yanyan Zhang, Cheng Zhu, Wentao Xu, Xiaolei Hu, Domingo Antonio Sánchez Martínez, José Luis Alonso Romero, Ming Yan, Ying Dai, Hua Wang

**Affiliations:** 1 Department of Medical Oncology, The First Affiliated Hospital of Anhui Medical University, Jixi Road 218, Hefei, 230022 China; 2 Breast Center, Department of General Surgery, Nanfang Hospital, Southern Medical University, Guangzhou, 510515 China; 3 Department of Medical Oncology, Clinical University Hospital Virgen Arrixaca, Murcia, 30120 Spain; 4 Department of Medical Oncology, The Second Affiliated Hospital of Anhui Medical University, Hefei, 230022 China

**Keywords:** Triple-negative breast cancer, Peripheral blood cell count, Anti-PD-1, Immunotherapy, Immune checkpoint inhibitors

## Abstract

**Background:**

Immune checkpoint inhibitors (ICIs) can improve survivals of metastatic triple negative breast cancer (mTNBC); however, we still seek circulating blood biomarkers to predict the efficacy of ICIs.

**Materials and methods:**

In this study, we analyzed the data of ICIs treated mTNBC collected in Anhui Medical University affiliated hospitals from 2018 to 2023. The counts of lymphocytes, monocytes, platelets, and ratio indexes (NLR, MLR, PLR) in peripheral blood were investigated via the Kaplan-Meier curves and the Cox proportional-hazards model.

**Results:**

The total of 50 mTNBC patients were treated with ICIs. High level of peripheral lymphocytes and low level of NLR and MLR at baseline and post the first cycle of ICIs play the predictable role of immunotherapies. Lymphocytes counts (HR = 0.280; 95% CI: 0.095–0.823; *p* = 0.021) and NLR (HR = 1.150; 95% CI: 1.052–1.257; *p* = 0.002) are significantly correlated with overall survival. High NLR also increases the risk of disease progression (HR = 2.189; 95% CI:1.085–4.414; *p* = 0.029). When NLR at baseline ≥ 2.75, the hazard of death (HR = 2.575; 95% CI:1.217–5.447; *p* = 0.013) and disease progression (HR = 2.189; 95% CI: 1.085–4.414; *p* = 0.029) significantly rise. HER-2 expression and anti-tumor therapy lines are statistically correlated with survivals.

**Conclusions:**

Before the initiation of ICIs, enriched peripheral lymphocytes and poor neutrophils and NLR contribute to the prediction of survivals.

**Supplementary Information:**

The online version contains supplementary material available at 10.1186/s12905-023-02871-6.

## Background

Triple-negative breast cancer (TNBC) accounts for around 15% of breast cancers. The prognosis of TNBC is unfavorable due to poor differentiation, strong invasion, and easy recurrence, resulting in 5-year survival less than 30% in metastatic stage [1]. A fraction of mTNBC patients responded to immune checkpoint inhibitors (ICIs) monotherapy or combined treatments. Thus, the selection of ICIs beneficial subgroups and how to improve the efficacy of ICIs in mTNBC are still challenging.

High levels of PD-L1 and stromal tumor-infiltrating lymphocytes (TILs) reflect the potential benefit of ICIs in mTNBC [2, 3]. Mesenchyme TILs enrichment contributed to reduced relapse and longer survival in TNBC [4]. Dieci et al. reported that the five-year overall survival (OS) of high TILs group in neoadjuvant thermotherapy was 91%, in contrast to low TILs group (55%) [5]. However, the distribution of TILs varied significantly, depending on tumor heterogeneity [6]. TILs score access is also limited due to the test availability and high expense in hospital. Peripheral blood indices were reported to predict effects of ICIs in non-small cell lung cancer and early-stage hepatocellular carcinoma, highlighting the need to develop circulating biomarkers to foresee recurrence risk in mTNBC [7, 8].

Our study proved the correlation between circulating blood cells and the therapeutic efficacies of ICIs in mTNBC.

## Material and methods

### Patient population

mTNBC patients treated with ICIs in affiliated hospitals of Anhui Medical University from 2018 to 2023 were collected and screened. They were administered with ICIs and/or chemotherapies. Tumor evaluation by CT (computed tomography) scanning was performed after every two cycles of treatment according to RECIST 1.1 (Response Evaluation Criteria in Solid Tumors version 1.1). Our study was approved by the Ethics Committee of Anhui Medical University (Reference number. PJ 2023-11-58).

### Treatment and data collection

A total 83 mTNBC patients were collected and 50 patients treated with ICIs were included. The baseline features are listed in Table 1.

**Table 1:** Baseline characteristics

Patient Characteristics	Total (*N* = 50)
Age, median(range)	54(38–72)
Prior chemotherapy lines, No.(%)	
0	24(48%)
1	6(12%)
≥ 2	20(40%)
Optimal therapeutic effect, NO(%)	
PD	10(20%)
SD	26(52%)
PR	11(22%)
CR	3(6%)
HER-2 expression.NO(%)	
0	30(60%)
1+/2+	20(40%)
Hepatic metastases.NO(%)	
Yes	12(24%)
No	36(76%
Brain metastases.NO(%)	
Yes	7(14%)
No	43(86%)
Lung metastasis.NO(%)	
Yes	28(56%)
No	22(44%)
Chest wall metastasis.NO(%)	
Yes	24(48%)
No	26(52%)
Bone metastases.NO(%)	
Yes	23(46%)
No	27(54%)
Immune adverse effects.NO(%)	
Yes	13(26%)
No	37(74%)

The peripheral blood cell counts at baseline and prior to second-line treatments included white blood cell (WBC), absolute neutrophil (ANC), absolute lymphocyte (ALC), absolute monocyte (AMC) and blood platelet (PLT). NLR (ANC/ALC ratio), MLR (monocyte/lymphocyte ratio), and PLR (hemocyte/lymphocyte ratio) were calculated.

Tumor evaluation was performed post every two cycles of treatment; Adverse events were assessed with Immune-related Response Evaluation Criteria in Solid Tumors.

### Statistical analysis

Patient features were described via descriptive statistics. Overall survival (OS) and progression-free survival (PFS) were collected and analyzed. The Cox proportional risk model was established with hazard ratios (HRs) and 95% confidence intervals (CIs). The multi-variable death model was modified based on age (initial diagnosis) and therapeutic line numbers (0, 1, 2, 3 and higher). All statistical tests were two-sided with significance threshold (alpha, α) at 0.05.

## Results

### Patient characteristics

Among a total of 83 mTNBC patients, 50 cases were selected, which received at least two cycles of ICIs. The baseline features are listed in Table 1. The median age was 54 years old and over half (*n* = 26, 52%) had received at least one line of palliative chemotherapy before immunotherapy. Low HER-2 expression is defined by 1 + to 2 + with absence of HER-2 amplification via fluorescence in situ hybridization (FISH). 40% of mTNBC are HER-2 lowly expressed (*n* = 20) and the disease control rate (DCR) by ICIs was 80%. The median OS (mOS) was 226 days and the median PFS (mPFS) was 145 days.

### The baseline and post-ICIs peripheral blood biomarkers in mTNBC

The mean baseline peripheral blood lymphocyte (PBLC) of ICI responding mTNBC subgroup (SD, PR and CR post immunotherapy) was 1.242*10^9^/L (95%CI:1.125–1.359), significantly higher than that in non-responding group, 0.925 *10^9^/L (95%CI: 0.0634–1.215) (*P* = 0.021). After one cycle of ICIs, the mean PBLC values in both groups were 1.258*10^9^/L (95%CI:1.137–1.380) *verus* 0.839*10^9^/L (95%CI: 0.6014–1.077) (*P* = 0.002). The NLR and MLR (2.30 [1.64–3.67] and 0.25 [0.17-0.0.32]) in beneficial group also decreased significantly, in contrast to those in ICI failed group (4.78[2.21–8.88] and 0.37[0.27–0.62]) (NLR: *P* = 0.018 and MLR: *P* = 0.023). The baseline monocyte counts and PLR were not found to significantly correlate with the response of ICIs.

### The correlation of peripheral blood biomarkers with immunotherapy outcomes

**Fig. 1:**
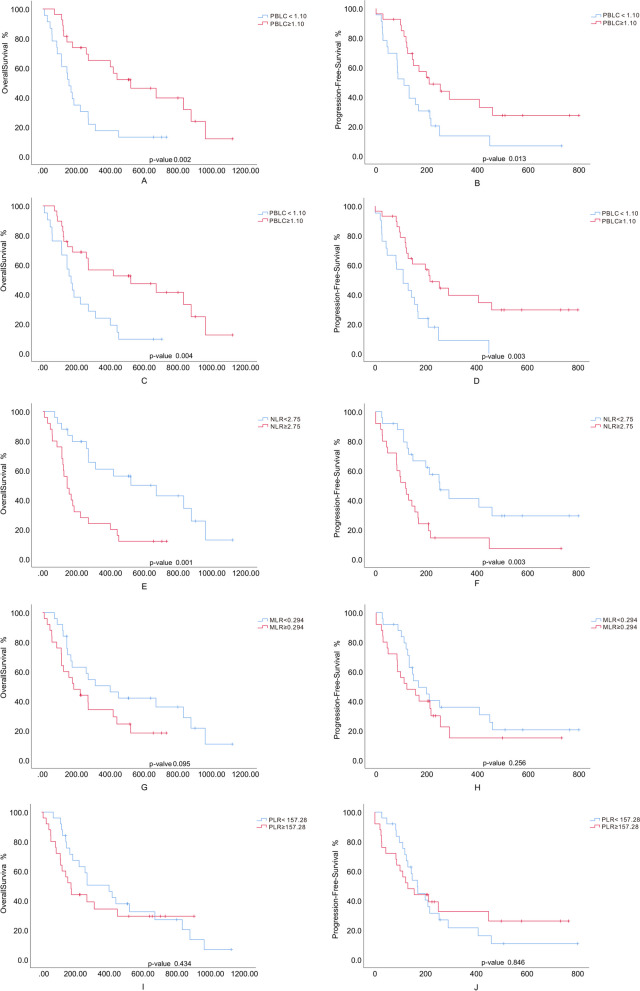
Kaplan-Meier survival curves for OS and PFS in mTNBC treated with ICIs. OS is plotted in (**A**, **C**, **E**, **G**, **I**) and PFS is plotted in (**B**, **D**, **F**, **H**, **J)**. Time is represented in days from initiation of ICIs. **A** and **B** patients are stratified by PBLC (prior to ICIs). Blue lines: PBLC (prior to ICIs) < 1.10*10^9^/L; red lines: PBLC (prior to ICIs) ≥ 1.10*10^9^/L. **C** and **D** patients are stratified by PBLC (post ICIs). Blue lines: PBLC (post ICIs) < 1.10*10^9^/L; red lines: PBLC (post ICIs) ≥ 1.10*10^9^/L. **E** and **F** patients are stratified by ANC/ALC ratio (NLR). Blue lines: NLR < 2.75; red lines: NLR ≥ 2.75. **G** and **H** patients are stratified by MLR. Blue lines: MLR < 0.294; red lines: MLR ≥ 0.294. **I** and **J** patients are stratified by PLR. Blue lines: PLR < 157.28; red lines: PLR ≥ 157.2

**Fig. 2:**
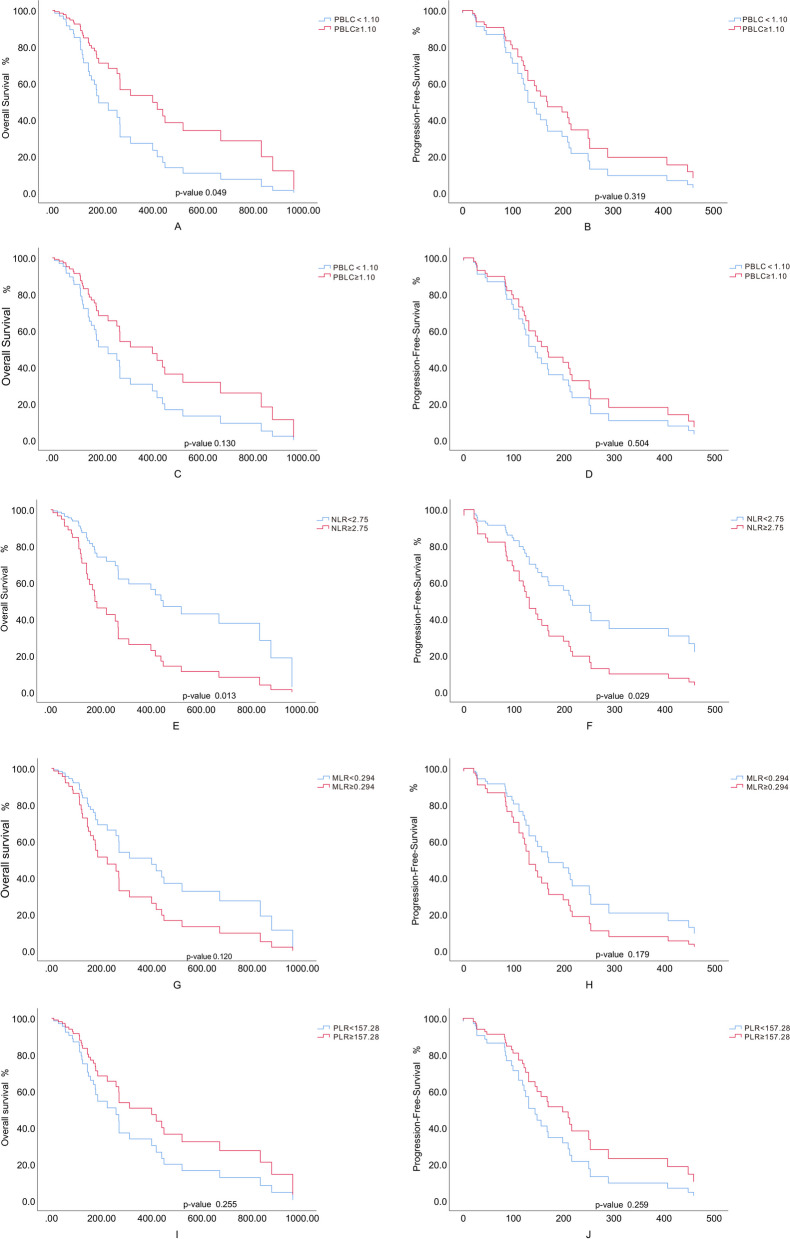
Cox proportional hazards model for OS and PFS of TNBC treated with ICIs. OS (**A**, **C**, **E**, **G**, **I**) and PFS (**B**, **D**, **F**, **H**, **J**) were plotted by COX proportional model in mTNBC. Time is presented as days from the start of immunotherapy. **A** and **B** patients are stratified by PBLC (prior to ICIs). Blue lines: PBLC (prior to ICIs) < 1.10*10^9^/L; red lines, PBLC (prior to ICIs) ≥ 1.10*10^9^/L. **C** and **D** patients are stratified by PBLC (post ICIs). Blue lines: PBLC (post ICIs) < 1.10*10^9^/L; red lines: PBLC (post ICIs) ≥ 1.10*10^9^/L. **E** and **F** patients are stratified by NLR. Blue lines: NLR < 75; red lines: NLR ≥ 2.75. **G** and **H** patients are stratified by MLR. Blue lines: MLR < 0.294 and red lines: MLR ≥ 0.294. **I** and **J** patients are stratified by PLR. Blue lines: PLR < 157.28 and red lines: PLR ≥ 157.28

Lymphocyte count reduction was defined as < 1.1*10^9^/L [9]. High PBLC significantly improved OS and PFS in mTNBC either in ICIs naïve cases or post-ICIs (Fig. 1). Based on adjusted treatment lines, age, liver metastasis and HER-2 expression, the baseline lymphocyte in ICI treated mTNBC was associated with OS (HR: 0.280; 95% CI: 0.095–0.823; *p* = 0.021). In the group with baseline lymphocyte over 1.10*10^9^/L (LN-high group), mOS was 520 days (95% CI: 207.8-832.2), and 12-month survival rate was 55.6%. The group with baseline lymphocyte less than 1.10*10^9^/L (LN-low group) showed that mOS was 155 days (95% CI: 117.4-192.6) (HR: 0.482; 95% CI: 0.233–0.999; *p* = 0.049), and the 12-month survival rate was 17.4% (*p* = 0.06) (Fig. 2). The 6-month PFS in both groups was 51.9% and 30.4%, but without statistical significance (*p* = 0.126). However, the 6-month PFS rate post one cycle of ICIs in LN-high group (55.2%) significantly exceeded that in LN-low group (23.8%) (*p* = 0.027).

The cutoff points of NLR and PLR were defined at median values of samples. NLR, other than PLR and MLR, significantly extended survivals of mTNBC when it is over 2.75 (Fig. 1 and Supplementary Figures 1 and 2). The treatment lines, age, andHER-2 expression were adjusted accordingly in the multivariable analysis. The baseline NLR was significantly associated with OS (HR:1.150; 95% CI:1.052–1.257; *p* = 0.002) and PFS (HR:1.086; 95% CI:1.002–1.177; *p* = 0.045) (Table 2). The cutoff point of NLR was 2.75. The mOS of NLR-high group (≥ 2.75) and NLR-low group was 143 days (95% CI: 92.4-193.6) and 520 days (95% CI: 110.8-929.2) (HR: 2.575; 95% CI: 1.217–5.447; *p* = 0.013) (Fig. 2E). The mPFS in both groups was 118 days (95% CI: 77.2-158.8) and 253 days (95% CI: 110.8-929.2) (HR: 2.189; 95% CI: 1.085–4.414; *p* = 0.029) (Fig. 2F). The 12-month survival rates were 24% and 52% (*p* = 0.041), while the 6-month PFS rates were 24% and 60% (*p* = 0.01).

The baseline PLR also showed a positive correlation with survival time after immunotherapy (*p* = 0.028) (Table 2). However, the inter-group differences were not significant (Fig. 2 and Supplementary Figures 1 and 2).

**Table 2:** Multivariable models of OS and PFS adjusted to prognostic factors

Biomarkers HR (95% CI), *n* = 50	Multivariable model of OS	*P*	Multivariable model of PFS	*P*
PBLC (prior to ICIs)	0.280(0.095–0.823)	0.021	0.413(0.141–1.213)	0.108
Adjusted PBLC (prior to ICIs)	0.482(0.233–0.999)	0.049	0.693(0.337–1.426)	0.319
PBLC (post treatment)	0.459(0.148–1.422)	0.177	0.791(0.285–2.422)	0.681
NLR	1.150(1.052–1.257)	0.002	1.086(1.002–1.177)	0.045
Adjusted NLR	2.575(1.217–5.447)	0.013	2.189(1.085–4.414)	0.029
MLR	3.880(0.665–22.626)	0.132	3.433(0.610-19.327)	0.162
Adjusted MLR	1.802(0.858–3.787)	0.120	1.619(0.802–3.271)	0.179
PLR	1.004(1.000-1.008)	0.028	1.002(0.999–1.006)	0.161
Adjusted PLR	1.596(0.714–3.568)	0.255	1.596(0.709–3.593)	0.259
ICIs lines	1.929(1.289–2.887)	0.001	2.778(1.819–4.241)	0.00
Age	1.007(0.959–1.057)	0.784	1.075(1.011–1.143)	0.021

1. Age, HER-2 expression, and number of treatment lines and liver metastases were adjusted in PBLC, MLR and PLR multivariable models. 2. Age, HER-2 expression, and treatment lines were adjusted in NLR multivariable model. 3. Age, HER-2 expression, and liver metastasis were adjusted in ICIs lines multivariable models. 4. HER-2 expression, number of treatment lines, liver metastasis were adjusted in Age multivariable model

### HER-2 expression and anti-tumor therapeutic lines on the survival of ICI-treated mTNBC

HER-2 low expression is defined as HER-2 1 + or 2 + immunohistochemistry without gene amplification. With adjusted therapeutic lines, age, and liver metastasis, HER-2 expression in ICI-treated mTNBC was significantly associated with OS (HR:3.253; 95% CI,1.418–7.460; *p* = 0.005) and PFS (HR:2.710; 95% CI:1.226–5.992; *p* = 0.014) (see Supplementary Figures 3 and 5). The median OS and 12-month survival rate in HER-2 low subgroup were 343 days and 50% respectively. The median PFS and 6-month survival rate in HER-2 low group were 206 days and 55%, superior to HER-2 zero group (mOS: 161 days; 12-month survival rate: 30%127; mPFS: 127 days; 6-month rate:33%). Metastatic TNBC patients previously treated with less than two anti-tumor therapeutic lines prior to ICIs showed better OS and PFS than those with over one or two lines (Table 2 and Supplementary Figures 7 and 8).

### Safety

A total of 13 patients (26%) had grade 3 to 4 immune-related adverse responses, leading to the cease of immunotherapy. No significance was shown in mTNBC with and without adverse immune events (AEs) on baseline demographics.

The most common grade 3 to 4 immune-related AEs (irAEs) included myositis/myocardial damage (*n* = 7, 53.8%), pneumonia (*n* = 3, 15.4%), myelosuppression (*n* = 2, 15.4%), abnormal liver function (*n* = 1, 7.7%), and skin reaction (*n* = 1, 7.7%). ICIs were stopped immediately upon the occurrence of AEs; glucocorticoids and/or immunoglobulins were administrated accordingly. Four cases died of irAEs (three cases of cardiac dysfunction and one case of myelosuppression). The baseline peripheral lymphocyte count, and monocyte count declined AE population (lymphocytes: *p* = 0.042, monocytes: *p* = 0.040).

## Discussions

In our study, absolute baseline lymphocyte enrichment improves the survival of mTNBC, which was further reflected even post one cycle of ICIs. In non-small cell lung cancer treated with ICIs, OS was prolonged with higher absolute baseline lymphocyte [10]. The relatively high lymphocyte count in peripheral blood is related to prolonged survival in gynecologic malignancies [11]. Anosheh et al. also reported less death risk in early-staged TNBCs, with higher absolute lymphocyte counts [12]. ICIs reverse the effects of PD-1 on lymphocyte signal conduction via PD-1–PD-L1 axis blockage, which facilitates the production of effective T cells and memory cells, inhibits the differentiation of TEX and T-Reg cells, and strengthens anti-tumor T-cell activation [13]. It is difficult to induce anti-tumor effects in absence of lymphocytes.

Additionally, higher PBLC contributes to better OS. Although baseline lymphocyte counts in ICIs naïve mTNBCs are generally higher than in those exposed to second- or third-line ICIs, the positive correlation between PBLC and OS is significant by statistical adjustment. Mechanistically, advanced metastatic breast cancer shows stronger immune suppression with insufficient TILs in cancer tissues [14, 15]. Previous studies prove a correlation between TILs and absolute lymphocyte count in breast cancer [16].

We also found that NLR negatively correlated with OS and PFS, in contrast to positive correlation with OS by PLR. NLR and PLR are predictive of poor prognosis in many types of tumors [17]. In the latest bioinformatic analysis involving 2,994 patients, TNBC with less genetic NLR were enriched in several immunity-related gene sets; TNBC carrying lower NLR might benefit from ICIs [18]. Tumor derived platelets recruit hemocyte and immune cells for migration and established inflammatory tumor microenvironment at primary and metastatic sites [19]. Inflammatory environment via high NLR impaired the clinical efficacy of ICIs and chemotherapy.

mTNBCs with baseline NLR less than 3.16 showed prolonged OS and PFS post neoadjuvant chemotherapies [20]. In non-small cell lung cancer with baseline NLR > 5.9, the therapeutic effect and long-term prognosis of anti-PD-1 inhibitors fell significantly [21]. In advanced gastric cancer and liver cancer, The NLR cutoff values were 3.23 and 3, respectively [22]. The values of NLR and PLR in our study were 2.75 and 157.28 (median), respectively. There are no commonly recommended thresholds of NLR etc. to predict the immunotherapeutic efficacy in cancer diseases, partially due to varied tumor immune microenvironments. Studies focusing on ICI predictors were mainly limited to non-small cell lung cancer and melanoma [23, 24]. In studies of pan-cancer species, breast cancer patients with high NLR benefited less from ICI clinically. The OS and PFS with low NLR or high tumor mutation loads increased partially post immunotherapy, but without statistical significance and pathological stratification [25]. Peripheral blood PLR and NLR in TNBC may be positively correlated with PD-L1 expression in immune cells, but without available pathological evidence [26]. Thus, the correlation of NLR and PLR with prognosis in breast cancer treated with immunotherapy is still veiled.

The prognostic effect of HER-2 low expression on breast cancer is still controversial. HER-2 low group intends to possess smaller tumor size and lower Ki67 index. In one retrospective study of 3689 breast cancer patients, HER-2 low is not associated with OS in TNBC without tumor proliferation genetic variation. Menopausal status, histological grade, Ki67 scores and percentage of TILs showed no significance difference [27]. In Chinese breast cancer population (*n* = 772), no statistical significance was observed in pCR rate between mTNBC HER-2 low and HER-2 zero groups; however, in non-pCR groups, prognosis was significantly improved in HER-2 low, other than Her-2 zero group, consistent with our data (*n* = 50) [28]. The prognosis of HER-2 will be validated in expanded sample size cohort studies.

In terms of tumor microenvironment (TME), TILs were significantly less expressed in HER-2 low tumor tissue other than HER-2 zero sample, indicating that HER-2 zero group might benefit more from ICIs. Therefore, the TILs levels are not convincing to elaborate prognosis of HER-2 low subgroup. Further investigations on mapped oncogene network in TNBCs were also demanded. The prognostic value of HER-2 low was solely restricted in specific subtypes. T cell exhausting was assumed post multiple lines of chemotherapies, partially contributing to inferior efficacy of ICIs. Our study also proved better survival when ICIs were initiated as the first or second anti-tumor therapy line.

Our study was limited by small sample size in the retrospective scale, mainly in Han population. Moreover, different combination of chemotherapies with immunotherapies might affect the exhaustion of bone marrow and PBLCs at baseline. mTNBCs with lowly expressed HER-2 showed longer survivals by immunotherapy than HER-2 negative subgroup. None was treated with anti-HER-2 antibodies or ADC drugs.

## Conclusions

Our data suggested that the baseline PBLC, NLR, MLR and absolute lymphocyte counts post ICIs clinically predict efficacies of anti-PD-1 antibodies in mTNBCs. HER-2 low expression and early ICI involvement also improve the survivals of mTNBCs. The access to whole blood sample from TNBC are easier and mor convenient in clinical practice. These findings may assist ICI related risk stratification and prevent unnecessary toxicities for those benefiting less from ICIs. However, the further investigation is demanded in a large scale and prospective view.

### Supplementary Information

**Additional file 1: Supplementary Figure 1.** Forest plot of the prognostic effect of relevant variables on OS. HR are calculated using Cox proportional hazards regression models and presented with 95% CIs.

** Additional file 2: Supplementary Figure 2.** Forest plot of the prognostic effect of relevant variables on PFS. HR are derived using Cox proportional hazards regression models and presented with 95% CIs.

** Additional file 3: Supplementary Figure 3.** Cox proportional hazards model for OS TNBC treated with ICIs. OS was plotted by Cox proportional model in mTNBC. Time is presented as days from the start of immunotherapy. Patients are stratified by HER-2. Blue lines: HER-2 (-); red lines, HER-2 (1+/2+).

** Additional file 4: Supplementary Figure 4.** Hazards model for OS TNBC treated with ICIs. OS was plotted by hazards model in mTNBC. Time is presented as days from the start of immunotherapy. Patients are stratified by HER-2. Blue lines: HER-2 (-); red lines, HER-2 (1+/2+).

** Additional file 5: Supplementary Figure 5.** Cox proportional hazards model for PFS TNBC treated with ICIs. PFS was plotted by Cox proportional hazards model in mTNBC. Time is presented as days from the start of immunotherapy. Patients are stratified by HER-2. Blue lines: HER-2 (-); red lines, HER-2 (1+/2+).

** Additional file 6: Supplementary Figure 6.** Hazards model for PFS TNBC treated with ICIs. PFS was plotted by hazards model in mTNBC. Time is presented as days from the start of immunotherapy. Patients are stratified by HER-2. Blue lines: HER-2 (-); red lines, HER-2 (1+/2+).

** Additional file 7: Supplementary Figure 7.** Cox proportional hazards model for OS in TNBC treated with ICIs. OS was plotted by Cox proportional hazards model in mTNBC. Time is presented as days from the start of immunotherapy. Patients are stratified by ICI lines. Blue lines: 1st line; red lines: 2nd line; green line: ≥ 3rd line.

** Additional file 8: Supplementary Figure 8.** Cox proportional hazards model for PFS in TNBC treated with ICIs. PFS was plotted by Cox proportional hazards model in mTNBC. Time is presented as days from the start of immunotherapy. Patients are stratified by ICI lines. Blue lines: 1st line; red lines: 2nd line; green line: ≥3rd line.

## Data Availability

All the data we used in this study were available as described in the “Material and methods” section.
