# Supplementary material for: Circulating blood biomarkers correlated with the prognosis of advanced triple negative breast cancer
Source: BMC Womens Health. 2024 Jan 13;24:38. doi: 10.1186/s12905-023-02871-6 (PMC10787989; doi:10.1186/s12905-023-02871-6)
Supplement: Supplementary file 1 — Additional file 1: Supplementary Figure 1. Forest plot of the prognostic effect of relevant variables on OS. HR are calculated using Cox proportional hazards regression models and presented with 95% CIs. [file 12905_2023_2871_MOESM1_ESM.pptx]

## Slide 1
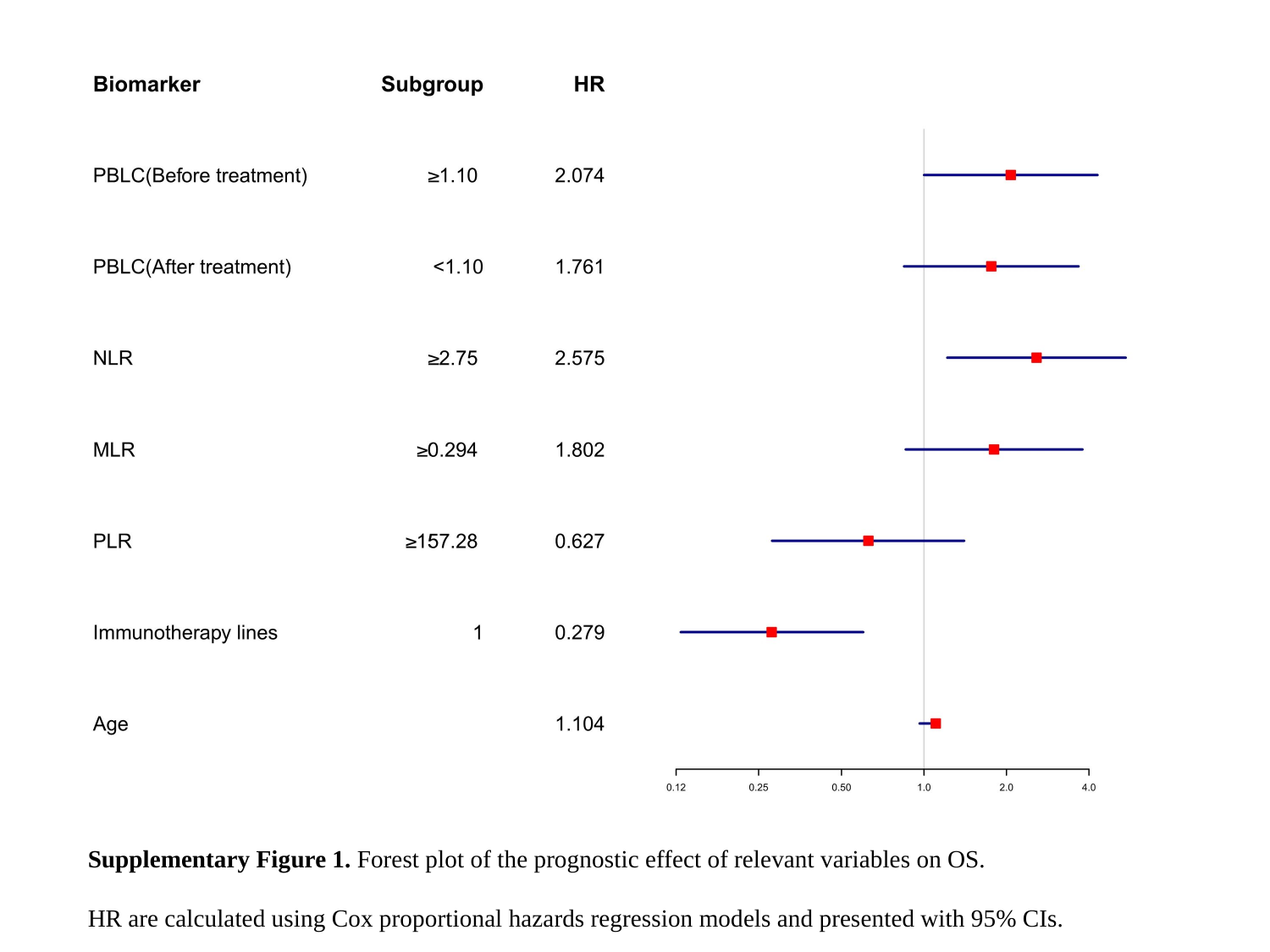

Supplementary Figure 1. Forest plot of the prognostic effect of relevant variables on OS.
HR are calculated using Cox proportional hazards regression models and presented with 95% CIs.
