# Supplementary material for: Circulating blood biomarkers correlated with the prognosis of advanced triple negative breast cancer
Source: BMC Womens Health. 2024 Jan 13;24:38. doi: 10.1186/s12905-023-02871-6 (PMC10787989; doi:10.1186/s12905-023-02871-6)
Supplement: Supplementary file 3 — Additional file 3: Supplementary Figure 3. Cox proportional hazards model for OS TNBC treated with ICIs. OS was plotted by Cox proportional model in mTNBC. Time is presented as days from the start of immunotherapy. Patients are stratified by HER-2. Blue lines: HER-2 (-); red lines, HER-2 (1+/2+). [file 12905_2023_2871_MOESM3_ESM.pptx]

## Slide 1
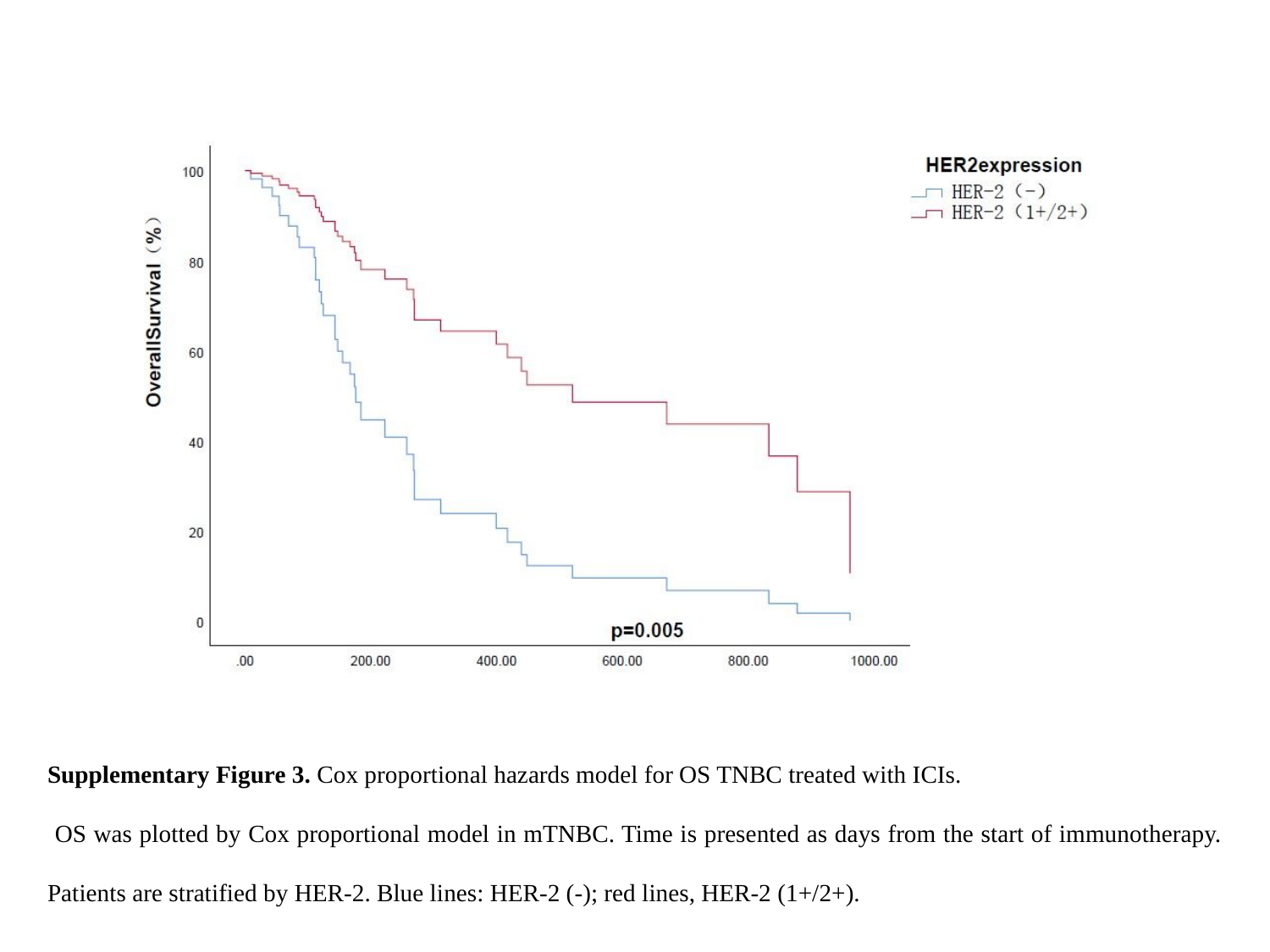

Supplementary Figure 3. Cox proportional hazards model for OS TNBC treated with ICIs.
 OS was plotted by Cox proportional model in mTNBC. Time is presented as days from the start of immunotherapy. Patients are stratified by HER-2. Blue lines: HER-2 (-); red lines, HER-2 (1+/2+).
.
