# Supplementary material for: Circulating blood biomarkers correlated with the prognosis of advanced triple negative breast cancer
Source: BMC Womens Health. 2024 Jan 13;24:38. doi: 10.1186/s12905-023-02871-6 (PMC10787989; doi:10.1186/s12905-023-02871-6)
Supplement: Supplementary file 4 — Additional file 4: Supplementary Figure 4. Hazards model for OS TNBC treated with ICIs. OS was plotted by hazards model in mTNBC. Time is presented as days from the start of immunotherapy. Patients are stratified by HER-2. Blue lines: HER-2 (-); red lines, HER-2 (1+/2+). [file 12905_2023_2871_MOESM4_ESM.pptx]

## Slide 1
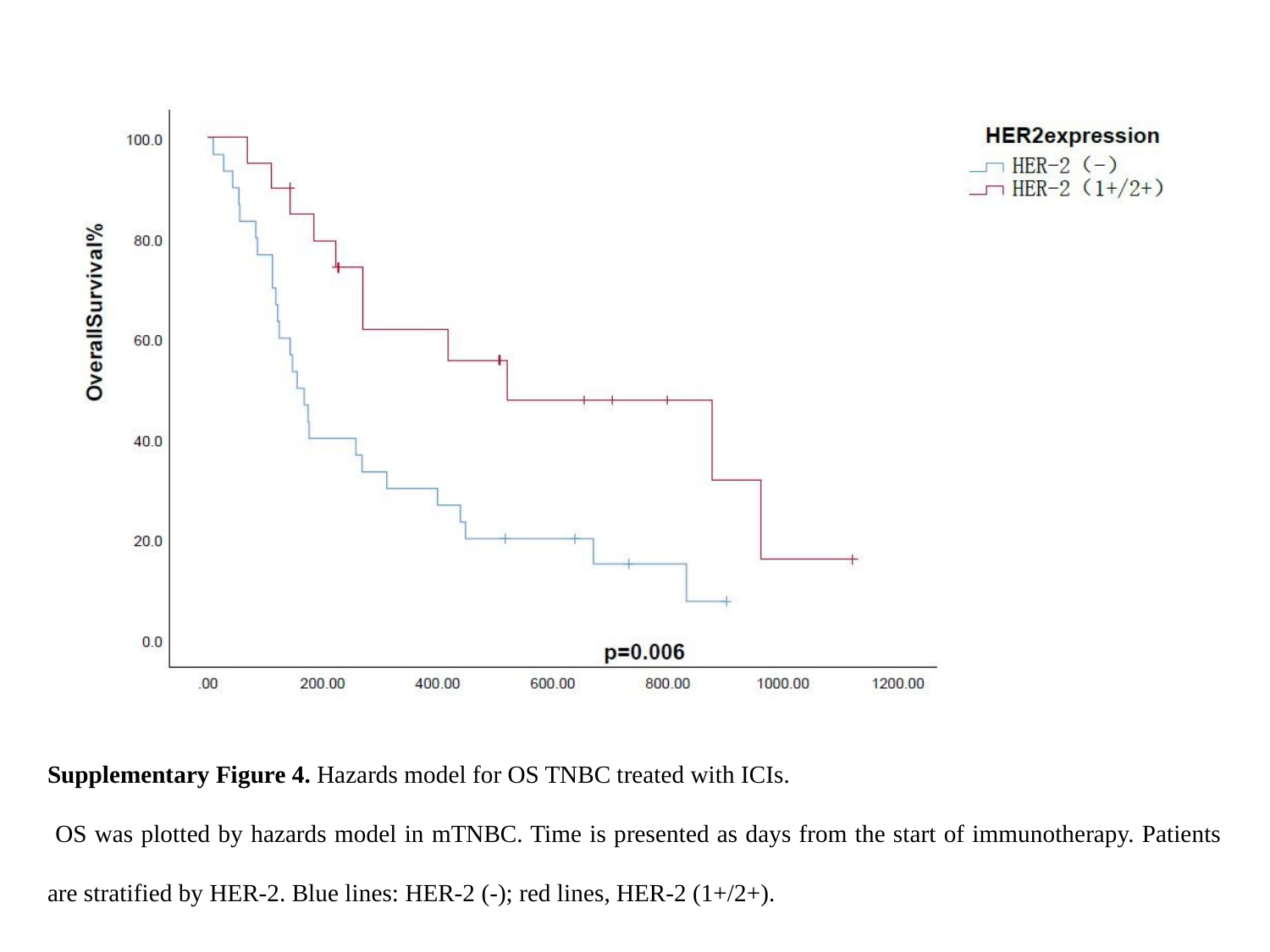

Supplementary Figure 4. Hazards model for OS TNBC treated with ICIs.
 OS was plotted by hazards model in mTNBC. Time is presented as days from the start of immunotherapy. Patients are stratified by HER-2. Blue lines: HER-2 (-); red lines, HER-2 (1+/2+).
.
