# Supplementary material for: Circulating blood biomarkers correlated with the prognosis of advanced triple negative breast cancer
Source: BMC Womens Health. 2024 Jan 13;24:38. doi: 10.1186/s12905-023-02871-6 (PMC10787989; doi:10.1186/s12905-023-02871-6)
Supplement: Supplementary file 5 — Additional file 5: Supplementary Figure 5. Cox proportional hazards model for PFS TNBC treated with ICIs. PFS was plotted by Cox proportional hazards model in mTNBC. Time is presented as days from the start of immunotherapy. Patients are stratified by HER-2. Blue lines: HER-2 (-); red lines, HER-2 (1+/2+). [file 12905_2023_2871_MOESM5_ESM.pptx]

## Slide 1
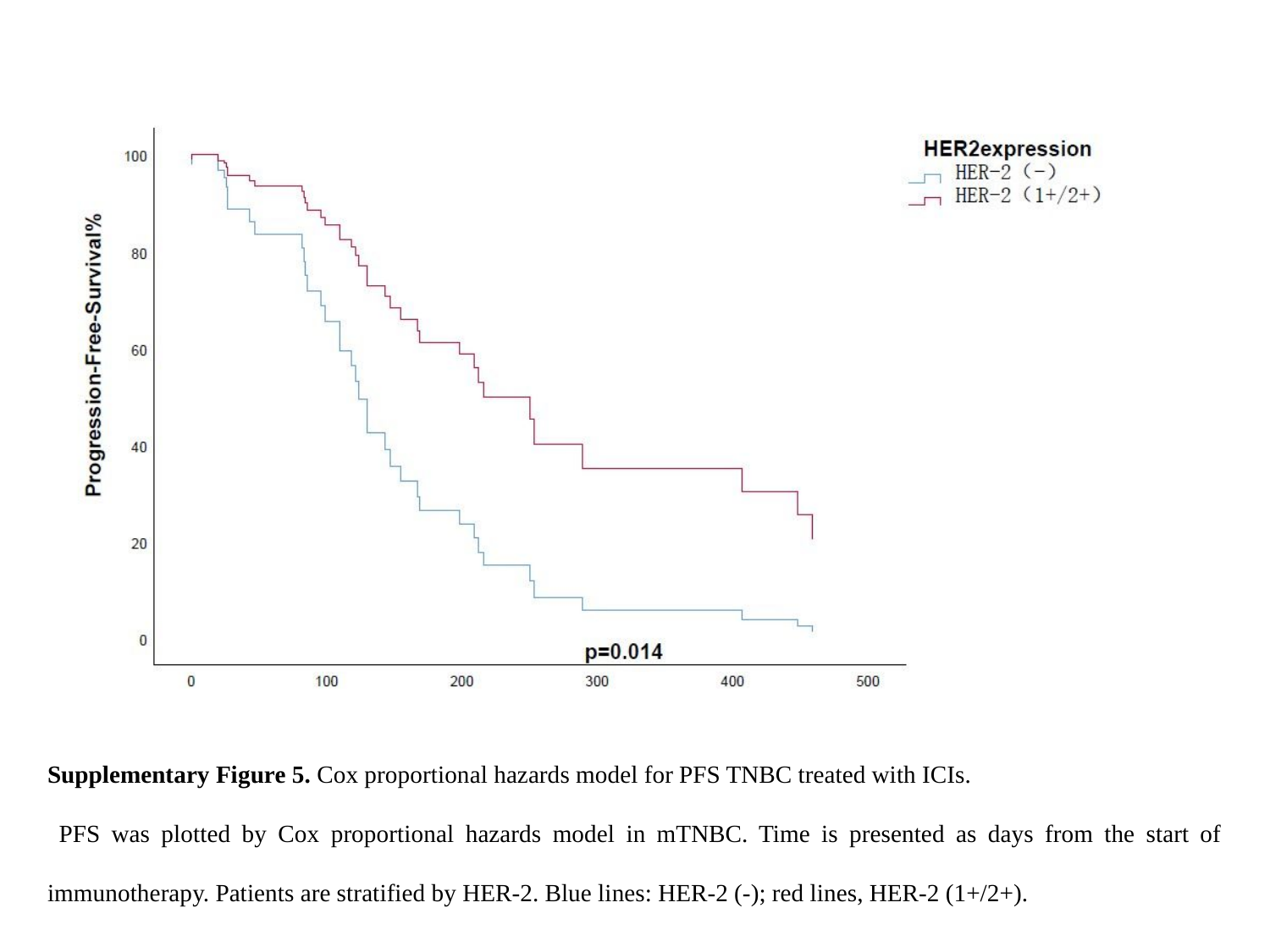

Supplementary Figure 5. Cox proportional hazards model for PFS TNBC treated with ICIs.
 PFS was plotted by Cox proportional hazards model in mTNBC. Time is presented as days from the start of immunotherapy. Patients are stratified by HER-2. Blue lines: HER-2 (-); red lines, HER-2 (1+/2+).
.
