# Supplementary material for: Circulating blood biomarkers correlated with the prognosis of advanced triple negative breast cancer
Source: BMC Womens Health. 2024 Jan 13;24:38. doi: 10.1186/s12905-023-02871-6 (PMC10787989; doi:10.1186/s12905-023-02871-6)
Supplement: Supplementary file 7 — Additional file 7: Supplementary Figure 7. Cox proportional hazards model for OS in TNBC treated with ICIs. OS was plotted by Cox proportional hazards model in mTNBC. Time is presented as days from the start of immunotherapy. Patients are stratified by ICI lines. Blue lines: 1st line; red lines: 2nd line; green line: ≥ 3rd line. [file 12905_2023_2871_MOESM7_ESM.pptx]

## Slide 1
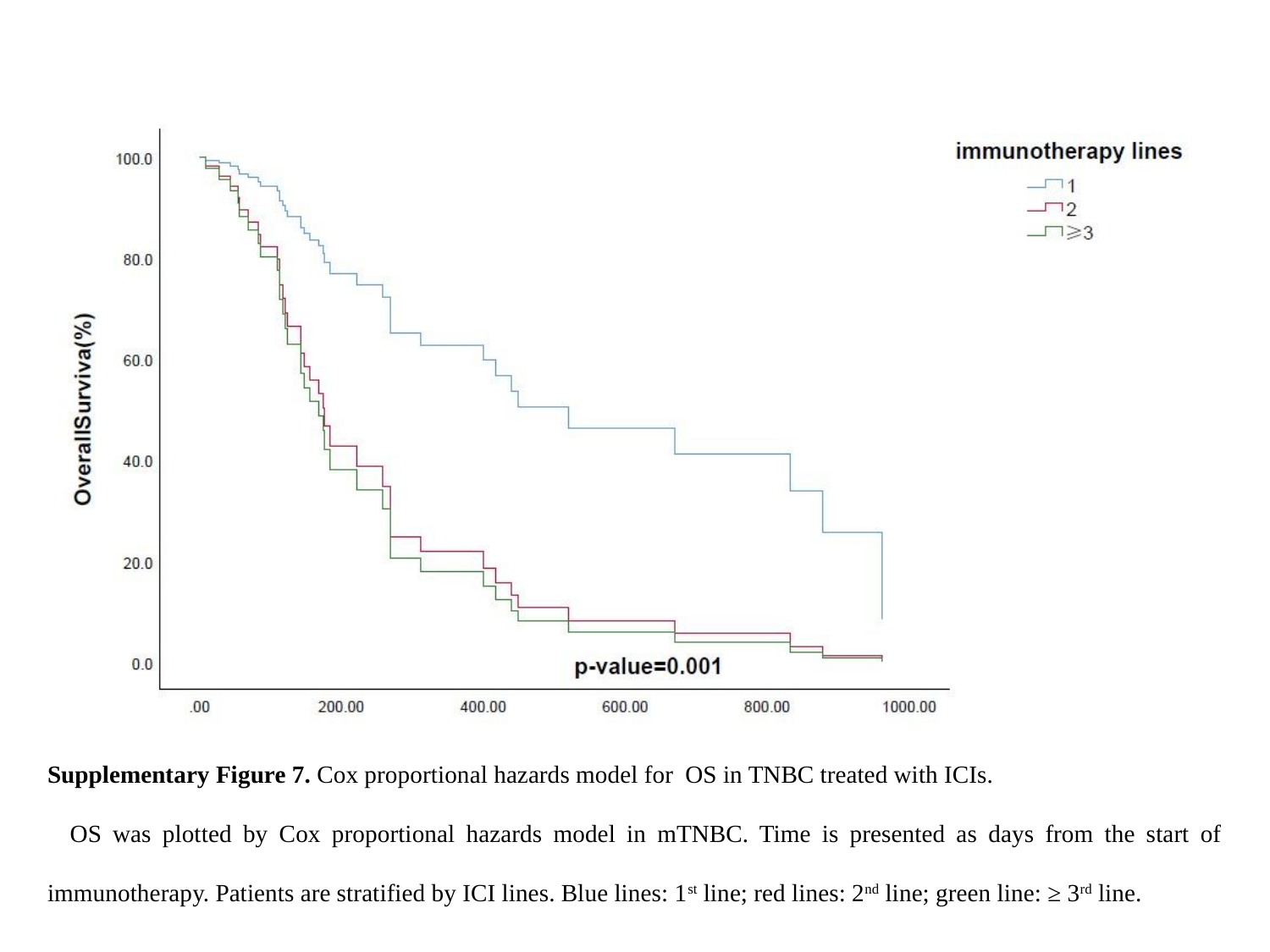

Supplementary Figure 7. Cox proportional hazards model for OS in TNBC treated with ICIs.
 OS was plotted by Cox proportional hazards model in mTNBC. Time is presented as days from the start of immunotherapy. Patients are stratified by ICI lines. Blue lines: 1st line; red lines: 2nd line; green line: ≥ 3rd line.
.
